# Advances in Chemotherapy and Targeted Therapies in Endometrial Cancer

**DOI:** 10.3390/cancers14205020

**Published:** 2022-10-14

**Authors:** Setsuko K. Chambers

**Affiliations:** Department of Obstetrics and Gynecology, Division of Gynecologic Oncology, The University of Arizona Cancer Center, University of Arizona, Tucson, AZ 85724, USA; schambers@uacc.arizona.edu

**Keywords:** endometrial cancer, molecular subtyping, targeted therapy

## Abstract

Endometrial cancer is now recognized to be several diseases with differing biology and responses to treatment. Improved molecular characterization has furthered the development and testing of targeted therapies in the different cohorts of endometrial cancer. Lessons are being learned from other cancers that share similar molecular typing, and hence, potentially similar tumor behavior. This commentary serves as a broad overview of the types of advances to which our patients now have access.

## 1. Introduction

Endometrial cancer is not one disease; hence, the treatment has evolved to be both different and, interestingly, more encompassing, at the same time. Clinically we have divided endometrial cancer into two large classes: one is the more common low-grade endometrioid endometrial carcinoma, and the other includes a histologically mixed group of high-grade endometrial malignancies including endometrial serous carcinoma, clear cell carcinoma, uterine carcinosarcoma and high-grade endometrioid or undifferentiated carcinomas.

## 2. Molecular Classifications

**Figure 1:**
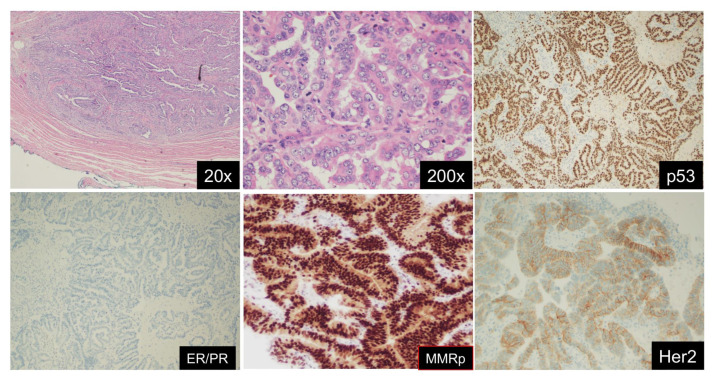
Endometrial serous carcinoma (ESC) with its classic immuno- and molecular profile. ESC shows deep myometrial invasion (**upper left**) and striking nuclear atypia in a high-power view (**upper mid**). Immunohistologically, the tumor shows mutant p53 (**upper right**), loss of ER/PR expression (**lower left**), proficient MMR panel (**lower mid**), and 3+ Her2/neu overexpression. By sequencing analysis, the tumor shows no POLE ultramutation (not shown). Molecular classification: TCGA group 4, p53 aberrant type.

However, based on the TCGA genomic profiling, four distinct molecular groups have been identified: DNA Polymerase epsilon ultra-mutated classification which portends a good prognosis, microsatellite instability hypermutated (intermediate prognosis), copy number-low, and copy number-high (latter which includes p53 mutations, the worst prognosis) [1]. A more simplified, and now validated molecular classifier, ProMisE, helps clinically to account for some of the types of tumors which we can target today [2,3] (Figure 1). Mismatch-repair (MMR)-deficiency is evaluated with immunohistochemistry (IHC). The Polymerase ε (*POLE*) exons 9–14 are sequenced for mutations. Lastly, IHC for p53 is performed to determine normal (wildtype) expression versus complete loss/null or overexpressed. However, broader molecular analysis of endometrial carcinoma tissue is not only useful but is now common practice for the determination of medical therapy in this disease. Papers have focused on targeted therapies for endometrial serous and clear cell carcinomas [4,5]. As the examples which follow show, the study of Her2/neu expression with or without amplification, ARID1a mutation, and ER/PR IHC status, among others such as tumor mutational burden, constitute critical actionable findings as well.

## 3. Standard Chemotherapies

The backbone of combination chemotherapy in endometrial cancer is carboplatinum and paclitaxel; this standard of care is widely used in all endometrial cancers. Bevacizumab can be considered in combination with these two agents. Other chemotherapeutic agents include anthracyclines or cisplatinum, ifosfamide combinations for carcinosarcoma, or trabectedin for uterine leiomyosarcoma [6].

## 4. Hormonal Management

Low-grade endometrioid carcinomas are almost always ER+ PR+ and, thus, are frequently managed with hormonal approaches. Further, these tumors frequently have PTEN or PI3-kinase pathway mutations. This would include the standard approaches such as megace, aromatase inhibitors, progestin secreting IUD’s such as the Mirena IUD; the latter is now frequently used in patients who are not good surgical candidates or those who are seeking retention of reproductive potential. Investigational therapies are also being studied, such as combinations of mTOR inhibitors (such as Everolimus) and hormonal therapy which have shown efficacy or targeting CDK4/6 in hormone receptor-positive endometrial cancers.

## 5. Deficient Mismatch Repair and Immuno-Oncology

Patients with genetic mutations consistent with Lynch syndrome have inherited a significant risk for endometrioid endometrial carcinomas, although there may be an association with endometrial serous carcinomas as well. These tumors are characterized by microsatellite instability (MSI-H) and deficient MMR, and frequently by high tumor mutational burden. Due to this characteristic, these tumors are commonly more sensitive than MMR-proficient tumors to immunotherapy, such as checkpoint inhibitors including pembrolizumab, and more recently nivolumab, dostarlimab, and avelumab [7]. Much more commonly, an epigenetic abnormality such as MLH1 promoter hypermethylation underlies the MSI-H and deficient MMR findings, characteristic of many endometrioid carcinomas of the endometrium, as well as the dedifferentiated endometrial carcinoma. One would assume that, regardless of mechanism (genetic or epigenetic), these tumors would prove equally sensitive to immunotherapy. However, recently there was a suggestion that the epigenetic mechanism may not lead to tumors which are consistently more sensitive to immunotherapy. This may be conjectured to be due to the higher likelihood of epigenetic dysregulation with reversion.

## 6. Proficient Mismatch Repair and Targeted Treatment

Clinical trials of advanced/recurrent endometrial cancer have led to the finding that the combination of pembrolizumab and lenvatinib (VEGFR inhibitor) has activity in endometrioid carcinomas, regardless of MSI status [8,9]. Findings also suggest the activity of pembrolizumab and lenvatinib in endometrial serous carcinoma. It should be noted, however, that this combination can have significant toxicity, thus, lower doses of lenvatinib have been examined. Clinical pathways are suggested for pre-emptive management of toxicity prevention.

## 7. Her2/neu Targeted Therapy Success

While many clinical trials have even recently excluded carcinosarcomas as an eligibility criterion, now it is more clearly understood that this entity should be considered high-grade (de-differentiated) [10], since both the epithelial component of carcinosarcomas and metastases/recurrence are frequently high-grade serous carcinomas. This entity should be included in all studies which include endometrial serous carcinoma and other high-grade endometrial cancers. This is a critical advance, as treatment specific for recurrent or advanced uterine carcinosarcomas is toxic and the patients are not curable.

For instance, the finding that the addition of trastuzumab to chemotherapy for high expressers of Her2/neu in endometrial serous carcinoma improved overall survival [11], has now been extended to other endometrial carcinomas, including carcinosarcomas. This is in contradistinction to the lack of expression and impact in high-grade serous ovarian cancers. In fact, studies of high-grade uterine cancer show that 17 to 30% have Her2/neu amplification and up to 61 to 80% express the protein. Thus, trastuzumab is being applied to carcinosarcomas and the 10% of endometrioid carcinomas that express her2/neu.

Notably, there is activity of anti-Her2/neu agents in Her2/neu low (IHC 1-2+) breast cancer cases. Due to this finding, a trastuzumab antibody-drug conjugate (T-DXd) was studied in uterine carcinosarcomas where there was a 54% objective response rate in Her2/neu high and 70% in Her2/neu low cases with a median overall progression-free survival of 6.7 months.

These types of critical findings have led to even further expansion with the application of trials which study many different Her2/neu-expressing tumors at once including both gynecologic and non-gynecologic carcinomas. An example is the DESTINY-pan Tumor 02 multi-cohort trial.

## 8. ARID1A Mutation Targeted Therapies

Other endometrial cancer studies focused on targeted therapies based on the results of these types of agents in ovarian epithelial carcinoma. Clear cell and endometrioid carcinomas of the ovary and endometrium, both frequently harbor ARID1A mutations, and hence, targeted therapy such as tazemetostat is being studied in these mutated tumors [12]. Studies are examining pathways active in clear cell carcinoma of the endometrium and the ovary as distinct from those active in renal clear cell carcinomas. This has led to multicohort studies that examine investigational agents in extra renal clear cell carcinomas. Of note, PDL+ tumor-infiltrating lymphocytes are dominant in the histologic subtype of clear cell carcinoma in general; thus, these tumors are sensitive to immuno-oncology approaches.

## 9. Search for a Role for PARP Inhibitors

PARP inhibitors are being tested in several ongoing trials (no results yet) which examine high-grade endometrial carcinomas including serous, clear cell, and carcinosarcomas, following the success seen in ovarian cancer. The rationale is that the genomic profile of p53+ leading to genomic instability in endometrial serous carcinoma resembles that of triple-negative breast cancer, and high-grade serous ovarian cancer [13]. This may predict a response of this subset of endometrial carcinomas to PARP inhibitors. Studies of PARP inhibitors in combination with anti-Her2/neu therapies, checkpoint inhibitors, and/or other targeted therapies, are seen in recurrent disease as well as up-front with chemotherapy in clinical trials.

Lastly, wee1 inhibitors active in synthetic lethality in p53 mutant high-grade serous ovarian carcinoma are being translated to other p53 mutant gynecologic tumors, such as endometrial serous carcinoma [14].

## 10. Conclusions

In conclusion, there is a large wave of ongoing clinical trials of targeted therapies and their combinations in endometrial cancer. This is spurred on by the advances in our understanding of the mutational and transcriptional landscape of endometrial cancer, and our willingness to expand beyond the traditional clinical understanding of the classifiers of endometrial cancer, to embrace other gynecologic and non-gynecologic cancers which have the same molecular abnormalities. Studies of rare tumors benefit greatly from this improved understanding. That what was thought to be rare is not so rare after all.
